# 283. Post COVID-19 syndrome and quality of life impairment in a cohort of severe COVID-19

**DOI:** 10.1093/ofid/ofac492.361

**Published:** 2022-12-15

**Authors:** Carla M Roman-Montes, Guillermo A Guaracha-Basañez, Yesenia C Flores-Soto, Maria Fernanda Gonzalez, Luis Alfredo Ponce-de-leon, Karla M Tamez-Torres, José Sifuentes-Osornio

**Affiliations:** INCMNSZ, Mexico, Distrito Federal, Mexico; INCMNSZ, Mexico, Distrito Federal, Mexico; INCMNSZ, Mexico, Distrito Federal, Mexico; INCMNSZ, Mexico, Distrito Federal, Mexico; INCMNSZ, Mexico, Distrito Federal, Mexico; INCMNSZ, Mexico, Distrito Federal, Mexico; INCMNSZ, Mexico, Distrito Federal, Mexico

## Abstract

**Background:**

Post-COVID-19 syndrome occurs usually 3 months from the onset of COVID-19 with symptoms that last for at least 2 months and cannot be explained by an alternative diagnosis (WHO definition, 2021). Our objective was to describe the prevalence, type, and duration of symptoms and their impact on the quality of life of patients hospitalized for COVID-19.

**Methods:**

A cross-sectional descriptive study was done, digital informed consent was obtained. Patients with a history of hospitalization for COVID-19 between March 2020 and October 2021 were invited to answer electronically an adaptation of the questionnaire for the identification of post-COVID-19 syndrome and the instrument EuroQol-5D (quality of life). Both were performed at 3, 6, 9 and 12 months after the initial evaluation for COVID-19.

**Results:**

We included 246 patients, of whom 76% (187/246) met the definition of post-COVID-19 syndrome, 54% were men, with a median age of 50 years (IQR 41-63). Sixty-three percent (117/187) of post-COVID-19 syndrome patients described a worse health status (OR 9.2, 95% CI 4.1-22.6, p=< 0.0001). The median time to symptoms onset after hospital discharge was 1 day (IQR 1-20), and the median duration of symptoms was 150 days (IQR 90-225). The most frequent symptoms were dyspnea 75% (141), arthralgia 71% (132), fatigue 68% (127), hair loss 60% (112), myalgia 53% (99), sleep disturbances 52% (97), dizziness 47% (88), and palpitations 41% (76) (Figure 1).

Regarding quality of life, the post-COVID-19 syndrome patients presented a lower visual analog scale of the EQ-5D versus the group without syndrome (80mm [IQR 70-90] vs. 89.5mm [IQR 75-90], p=0.05). All five dimensions of the quality of life were affected in the post-COVID-19 syndrome group; and dimensions of pain/discomfort, usual activities, and anxiety/depression showed a statistical difference (Fig 2).

**Figure 1.**
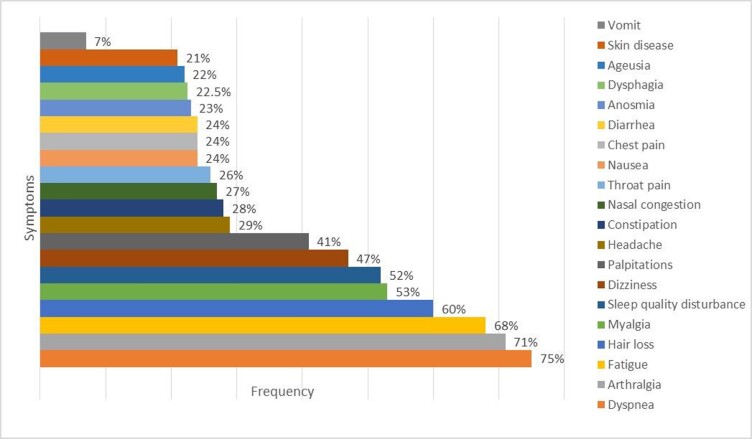
Frequency of symptoms in Post-COVID-19 syndrome.

**Figure 2.**
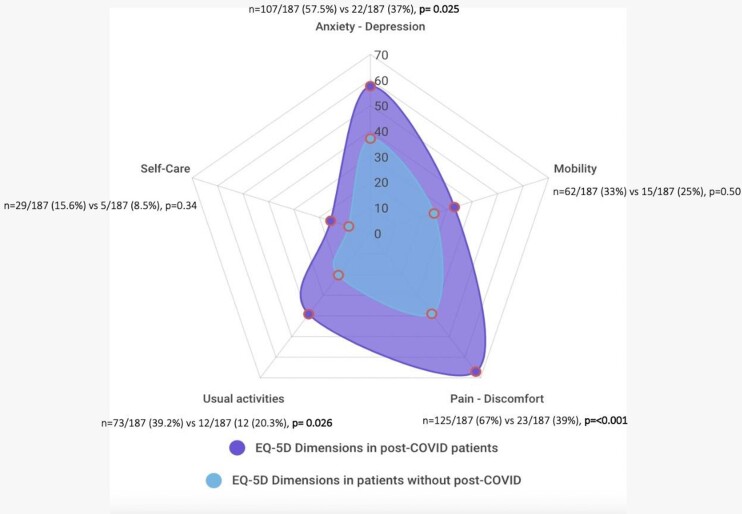
Distribution of the EQ-5D dimensions affected.

Euroqol (EQ)-5D:Specific instrument to describe and value health-related quality of life.

**Conclusion:**

Post-COVID-19 syndrome occurred in 76% of hospitalized patients, with prolonged duration and quality of life impairment. Dyspnea was the most frequent symptom. Timely diagnostic and therapeutic intervention is required.

**Disclosures:**

**All Authors**: No reported disclosures.

